# Familial essential thrombocythemia: 6 cases from a mono‐institutional series

**DOI:** 10.1002/ccr3.5525

**Published:** 2022-03-01

**Authors:** Vincenzo Accurso, Marco Santoro, Salvatrice Mancuso, Giorgia Vajana, Riccardo Tomasello, Cristina Rotolo, Giulia Camarda, Marta Mattana, Sergio Siragusa

**Affiliations:** ^1^ Hematology Division University Hospital Policlinico "Paolo Giaccone" Palermo Italy; ^2^ Hematology Unit Department of Health Promotion, Mother and Child Care, Internal Medicine and Medical Specialties (PROMISE) University of Palermo Palermo Italy

**Keywords:** essential thrombocythemia, familial, myeloproliferative neoplasms

## Abstract

Rarely essential thrombocythemia (ET) is diagnosed in more than one person within a family. Familial myeloproliferative neoplasms are underdiagnosed. In this report, we describe 6 couples of familial ET, evaluating the heterogeneity of the mutational state and the clinical presentation.

## Abstract 2

Familial essential thrombocythemia (ET) is very rare, also due to underdiagnosis. The collection of the cases could be useful to find out familial MPN diseases. Performing a complete blood count in relatives of patients with ET may lead to a suspicion of diagnosis of familial cases.

## 1 INTRODUCTION

Essential thrombocythemia (ET) is a chronic myeloproliferative neoplasm, characterized by marked thrombocytosis and significant thrombotic risk.^1^ In 2016, the WHO carried out a review on the case definition distinguishing major criteria (platelets >450*10^9^ per liter, bone marrow biopsy showing especially the expansion of the megakaryocyte line, the absence of criteria for the diagnosis of chronic myeloid leukemia, polycythemia vera, myelofibrosis, myelodysplastic syndromes, or other myeloid neoplasms, the presence of a pathogenic mutation of JAK2, CALR, or MPL) and minor criteria (presence of clonal markers and absence of evidence of reactive thrombocytosis). The diagnosis of ET requires either 4 major criteria or 3 major criteria and 1 minor criterion.^2^

Essential thrombocythemia is a relatively rare disease; recently in the USA has been reported an incidence of 1.1–2.0 cases per 100,000 inhabitants per year and a prevalence of 24–58 cases per 100,000 inhabitants. Compared to polycythemia vera and myelofibrosis, it has a better prognosis with a median survival of 13–23 years.^3^ In 50–60% of patients with ET, the JAK2 V617F point mutation can be revealed in the others, 15–30% showed the CALR mutation and in 1–5% the MPL mutation. Patients who do not have any of these driver mutations are defined as “triple‐negative”.^4^

When multiple cases of MPN are present in the same family group, these are called “familial MPN.” A study of 11,000 cases of MPN with 25,000 first‐degree relatives has detected that they have a 5–7 times higher risk of developing MPN.^5^ Some studies evaluate the frequency of familial MPN at 5%, other authors believe that the prevalence may be underestimated and evaluate it at 7.6%.^6^

There is evidence that TPO mutations may act as autosomal dominant character in determining ET in more than one person of a family through generations.^7^

In this article, we reported the clinical history and characteristics of 6 couples of familial essential thrombocythemia identified among the 269 consequent ET patients followed at our institution.

## 2 CASE 1

### 2.1 Patient A

A 52‐year‐old male patient with hypertension and dyslipidemia; the diagnosis was made in 2002 through a bone marrow biopsy; he reported myocardial infarction in 2001 and showed RBC 4,820,000/mmc, HGB 14.9 g/dl, PLT 720,000/mmc, and WBC 10,700/mmc. Abdomen ultrasound (US) did not reveal splenomegaly. The search for BCR‐ABL transcript was negative. Therapy with hydroxyurea and acetylsalicylic acid (ASA) was prescribed. Subsequently, in 2014, the search for driver mutations revealed “triple‐negativity.” In 2017, a new bone marrow biopsy confirmed the diagnosis of ET according to WHO 2016. The previous myocardial infarction places the patient at high risk according to the revised IPSET.^8^ The patient is currently in good health and continues the therapy with hydroxyurea and ASA.

### 2.2 Patient B

A 67‐year‐old male patient, brother of patient A, came to our observation in 2019, following recurrence of platelets elevation. He reports arterial hypertension, dyslipidemia, and a myocardial infarction happened in 2016. Investigations performed at the time of the diagnosis showed RBC 4850000/mmc, HGB 13.9 g/dl, PLT 534000/mmc, and WBC 10690/mmc. The presence of JAK2V617F mutation, with allelic burden 39.1% led to bone marrow biopsy that confirmed the diagnosis of ET according to 2016 WHO criteria. The patient is classified as high risk, according to the revised IPSET, and started therapy with hydroxyurea and ASA. Today he is in good health and continues to assume the same therapy.

## 3 CASE 2

### 3.1 Patient C

A 39‐year‐old woman came to our observation for the first time in 2010, following an occasional finding of elevated platelet counts.

The patient did not report any pathologies and appeared in good health conditions. Abdomen US revealed no splenomegaly. The investigations performed showed the presence of V617F mutation of JAK2, with 28% burden, RBC 4,400,000/mmc, HGB 13.1 g/dl, PLT 827,000/mmc, and WBC 7900/mmc. According to the revised IPSET score, the patient was classified as low risk. Follow‐up continues and appears healthy.

### 3.2 Patient D

A 38‐year‐old man, half‐brother of patient C (same father and different mother), came to our observation for the first time in 2016, due to the occasional finding of thrombocytosis. Objective examination was unremarkable. The complete blood count (CBC) revealed RBC 4,940,000/mmc, HGB 15.6 g/dl, PLT 933,000/mmc, and WBC 5720/mmc. The search for driver mutations showed the presence of the type2‐like mutation of exon 9 of the calreticulin gene. The diagnosis of ET is made after performing bone marrow biopsy. According to the revised IPSET score, he was classified as a very low risk. The patient does not assume any therapy or prophylaxis and continues the observation at our center with 3 to 6‐monthly control visits.

## 4 CASE 3

### 4.1 Patient E

A 39‐year‐old woman with obesity and dyslipidemia came to our observation for PLT elevation in 2007. On that occasion, CBC revealed RBC 4,260,000/mmc, HGB 13.6 g/dl, PLT 701,000/mmc, and WBC 9280/mmc. V617F mutation of JAK2 was negative, and the diagnosis of ET was performed on bone marrow biopsy histology. Since the patient referred cardiovascular risk factors, therapy with ASA 100 mg/day was recommended. Subsequently, in 2016, a further mutation research was performed for CALR and MPL genes, revealing wildtype alleles, and completing the diagnosis of triple‐negative ET. According to the revised IPSET scale, the patient was classified as very low risk. Currently, she is in good health and continues to assume anti‐aggregating therapy with ASA 100 mg a day.

### 4.2 Patient F

The 45‐year‐old female sister of patient E came to our observation for mild leukocytosis and platelet increase, in 2018, discovered at a CBC control done for persistent asthenia. She reported lower limb deep venous thrombosis, occurred in 2013 and treated with low molecular weight heparin for almost a month, hypertension in drug therapy, dyslipidemia, and obesity. CBC showed RBC 4,650,000/mmc, HGB 16.4 g/dl, PLT 525,000/mmc, and WBC 17,600/mmc. JAK2 mutations search resulted negative; subsequently also CALR, MPL, and the search for BCR‐ABL transcript were negative. The diagnosis of ET was performed by bone marrow histology in accordance with the 2016 WHO revision. The episode of DVT prior to MPN diagnosis recommended cytoreductive treatment that the patient is undergoing with hydroxyurea and ASA, with benefit on CBC and on MPN‐related symptoms.

## 5 DISCUSSION AND CONCLUSIONS

Although the identification of the driver mutations in JAK2, CALR, and MPL contributed significantly to the understanding of the molecular basis of MPN, many aspects still need to be clarified. The consideration that the same mutation can be related to three different diseases (ET, PV, and MF) arouses the plausible suspicion that other mechanisms may contribute to determining the phenotypic aspects of the pathologies. Several authors have hypothesized that the underlying germline parterre may predispose to the acquisition of oncogenic mutations.^9^

In this regard, Harutyunyan AS et al report the presence of the three different driver mutations in 3 subjects belonging to the same family.^10^ In fact, the role of some germline variants had already been reported. In particular, the JAK2 "GGCC" haplotype seems to significantly increase the risk to develop a JAK2‐mutated MPN.^11^ The same function seems to be performed by a germline variant in telomerase reverse transcriptase (TERT).^12^, ^13^

In the differential diagnosis and in the presence of a triple‐negativity, tests for the detection of germline TPO mutations that are associated with familial ET must be considered.^7^

Finally, in support of what has been reported so far, it is known that in JAK2‐mutated mice, the phenotypic manifestations are related to the mouse strain used, in fact, balb/c mice show marked leukocytosis and splenomegaly and medullary fibrosis compared to mice identified as C57B1/6.^14^, ^15^

Of note the fact that it seems there is any difference in clinical manifestation and prognosis between patients with sporadic and MPN and those with Familial MPN.^16^

The cases of ET we report, even if each couple presents the same histology, show extreme variability from the point of view of driver mutations, confirming what was reported by the various authors quoted in this report. Our series, however, represent only 2.6% of patients with ET.

Almost all authors agree that the cases of familial MPN are underestimated and underdiagnosed. However, it does not appear convenient on cost‐benefit ratio, to test *tout court* the relatives of patients with familial ET on molecular and histological screening; quite the opposite, in our opinion, it appears useful to perform a simple and inexpensive CBC on MPN relatives. This practice could select patients with suspect MPN, to be initiated for a complete MPN screening.

## CONFLICT OF INTEREST

All authors declare no conflict of interest.

## AUTHOR CONTRIBUTIONS

VA wrote the manuscript. MS and SM revised the draft. GV, RT, and CR collected the data. GC and MM prepared the draft. SS supervised the project.

## CONSENT

Written informed consent was obtained from the patient to publish this report in accordance with the journal's patient consent policy.

## Data Availability

The data used to support the findings of this study are available from the corresponding author upon request.
